# Long-term use of benzodiazepines in chronic insomnia: a European perspective

**DOI:** 10.3389/fpsyt.2023.1212028

**Published:** 2023-08-02

**Authors:** Michael Soyka, Imane Wild, Bérangère Caulet, Chrysoula Leontiou, Fabio Lugoboni, Göran Hajak

**Affiliations:** ^1^Department of Psychiatry and Psychotherapy, Ludwig Maximilian University, Munich, Germany; ^2^Idorsia Pharmaceuticals Ltd., Allschwil, Switzerland; ^3^Department of Internal Medicine, Addiction Unit, Verona University Hospital, Verona, Italy; ^4^University of Regensburg, Regensburg, Germany; ^5^Department of Psychiatry, Psychosomatic Medicine and Psychotherapy, Social Foundation Bamberg, Teaching Hospital of the University of Erlangen, Bamberg, Germany

**Keywords:** benzodiazepines, dependence, insomnia, sleep, therapy, opioids

## Abstract

Chronic insomnia occurs in ~10% of the general population and has numerous negative health effects. The recommended first line treatment of cognitive behavior therapy for insomnia is not widely available for patients in Europe, so pharmacotherapies such as benzodiazepine receptor agonist agents (benzodiazepines and Z-drugs) are commonly used. However, their use is only recommended for ≤4 weeks due to unproven long-term efficacy in treatment of chronic insomnia, and the risk of tolerance, and the potential for dependence and misuse. In Europe, recommendations limiting the use of benzodiazepines (lowest dose and shortest duration) in chronic insomnia are not always followed, likely due to the lack of approved effective alternative therapies. Here we present a recent pilot survey of the pharmacological treatment landscape in chronic insomnia in five European countries (France, Germany, Italy, Spain, and the United Kingdom) and physicians’ attitude toward treatment. The results suggest that benzodiazepines and Z-drugs are the most widely used treatments in chronic insomnia and are being used for longer than their recommended duration. Country variations in prescription rates were observed. Due to the known association between long-term benzodiazepine use and potential for developing dependence, further analysis of the literature was performed on the use and misuse of benzodiazepines. The results show that long-term use of benzodiazepines is associated with multiple consequences of treatment, including dependence, but also that previous use of benzodiazepines may increase the risk of opioid use disorder.

## 1. Introduction

Sleep is integral to the maintenance of key processes in the body, including energy conservation, metabolic waste clearance, memory consolidation, and the modulation of inflammation and immunity (1–3). Sleep is also recognized as one of eight essential components of cardiovascular health by the American Heart Association (4). Both sleep quantity and sleep quality are fundamental components of sleep (5).

Insufficient sleep is associated with numerous negative health effects and is a prevalent healthcare challenge worldwide (6), with the full clinical syndrome of chronic insomnia occurring in ~10% of the general population (7). The coronavirus disease 2019 (COVID-19) pandemic is reported to have driven an increase in the global prevalence of clinically significant insomnia to 16.7% (8). In Europe, prevalence estimates for chronic insomnia vary between countries (Table 1). Many patients with symptoms of insomnia lasting for years do not consult their physician and obtain a formal diagnosis (14, 15), suggesting that chronic insomnia is often under-reported.

**Table 1:** Prevalence of chronic insomnia reported in various European countries.

Country	Prevalence
Switzerland	11% of adults aged ≥18 years met DSM-5 criteria for chronic insomnia (9)
Germany	Prevalence of chronic insomnia was reported as 4% (10)
France	Chronic insomnia was reported in 15.8% of the population (11)
United Kingdom	Incidence of insomnia lasting ≥12 months was reported as 15% (12)
Italy	Chronic insomnia was reported in 13.2% of adults (13)

DSM-5, Diagnostic and Statistical Manual of Mental Disorders, Fifth Edition.

Psychiatric disorders are the most common comorbidities associated with insomnia (16) and insomnia can be a symptom or harbinger of other psychiatric disorders (17). It is estimated that 40% of all insomnia patients have a coexisting psychiatric condition (18, 19). Depression is the most common of these conditions and the presence of insomnia is considered a transdiagnostic symptom for depressive and anxiety disorders (20). Given the detrimental effects of insomnia on mental health, severity of daytime symptoms and recovery/remission of mental disorders, in patients with comorbid psychiatric illness and insomnia clinicians therefore need to address both conditions to achieve optimal treatment outcomes (21–23).

Chronic insomnia disorder is defined in the International Classification of Sleep Disorders, Third Edition (ICSD-3) as having sleep disturbance and associated daytime symptoms occurring at least three times per week, and present for at least 3 months (24). In the ICSD-3, short-term insomnia disorder is characterized by sleep/wake difficulties that fail to meet the minimal frequency and duration criteria of chronic insomnia disorder (24). The current version of the Diagnostic and Statistical Manual of Mental Disorders, Fifth Edition (DSM-5) adopts a similar approach to the ICSD-3 in specifying that those affected by chronic insomnia disorder have sleep difficulty occurring at least 3 nights per week, and this is present for at least 3 months, whereas acute−/short-term insomnia is defined as meeting the same diagnostic criteria, but with symptoms lasting less than 3 months (25).

Treatment guidelines for chronic insomnia include both non-pharmacological and pharmacological options (26, 27). At first line, cognitive behavioral therapy for insomnia (CBTi) is recommended in adults of any age by the European Sleep Research Society (ESRS) and in national guidelines for insomnia and sleep disorders for European countries (13, 27–29). However, in practice, use of CBTi appears to be limited in Europe. For example, in a survey by the European Insomnia Network (EIN) among 12 countries that are founding members of its CBTi Academy, only approximately 600 and 300 patients were estimated to have received CBTi annually in Norway and Italy, respectively (30). In France, it was estimated that only 15–30 centers (mostly academic hospitals with a sleep clinic) offered CBTi (30). The EIN highlighted that differences in healthcare systems across Europe do not represent a barrier to the availability of pill-based solutions, however the structure of health services and associated reimbursement mechanisms may play a part in “rationing” access to CBTi (30). They also noted that, because CBTi is traditionally delivered face-to-face, the shortage of training in this therapy represents an intrinsic limitation to the scalability of CBTi to meet population need and demand (30). In addition, although general practitioners (GPs) have a pivotal role in treating patients with insomnia, they rarely prescribe or are able to offer CBTi (30).

If CBTi is not deemed sufficiently effective, is not available, or is inaccessible to patients, pharmacological interventions–namely benzodiazepine (BZD) receptor agonists such as BZDs or Z-drugs (ZDs), and some sedating antidepressants–can then be offered (27). In insomnia, BZDs and ZDs are indicated for short-term use only and there are several BZD hypnotics available for this indication. Regulatory bodies in many countries, including France, Germany, Italy, Spain, and the United Kingdom, have issued guidance to regulate and limit the use of BZDs and other similar drugs to a maximum duration of 2–4 weeks due to the concerns linked with dependence, tolerance, and substance abuse (13, 27, 29, 31, 32). Positive long-term effects of BZDs are not proven (33).

Although multiple publications address the use of BZDs in chronic insomnia, quantitative data at the European level are lacking, and it is unclear whether the advice that “Long-term treatment of insomnia with BZ or BZRA is not generally recommended because of a lack of evidence and possible side-effects/risks” given by the ESRS 2017 guideline is being followed. This manuscript aims to explore the hypothesis that prolonged use of BZDs in the treatment of chronic insomnia may still be relatively common in Europe. We assessed the results of a series of pilot market research studies of the treatment landscape of chronic insomnia focused on five European countries (France, Germany, Italy, Spain, and the United Kingdom). This analysis included an examination of the duration of treatment with BZDs and ZDs in insomnia. Based on the resulting observations, we discuss some of the known and emerging awareness of risks associated with prolonged use of BZDs in the treatment of chronic insomnia. Where relevant, we also compare and contrast the information on BZDs with the data related to the use of ZDs. Within the literature, the risk profile of ZDs has not been characterized to the same depth as that for BZDs, so we have focused most of our discussions on the BZD class.

## 2. Methods

Pilot market research studies were commissioned by Idorsia to better understand and quantify the use of BZDs in insomnia in Europe. The methodology for these studies is described in brief below.

### 2.1. Primary research among healthcare professionals working in insomnia

This dataset comes from a primary market research (PMR) study conducted by Ipsos (Ipsos).^1^ Ethics committee approval was not required as Market Research as defined in Section 1 of the European Pharmaceutical Market Research Association (EphMRA) Code of Conduct does not require Clinical Research Ethics Committee or Independent Review Board approval (34). Data gathering involved a 30-min online questionnaire which was open to general practitioners (GPs), sleep specialists, neurologists, and/or psychiatrists involved in clinical management of people with insomnia. The questionnaire is included as supplementary material online.

The questionnaire was broadly divided into the following topic areas:

- Profiling questions, including work setting and numbers of years in practice
- Insomnia caseload, including number of patients seen and number of chronic versus acute insomnia patients
- Perception of insomnia, including how physicians perceive insomnia as a condition, how challenging they find it to manage and how much insomnia impacts on the lives of their patients
- Attitudes toward insomnia treatment and management, including perception toward different insomnia treatments covering different classes of prescriptions, and toward monitoring and follow-up of patients
- Treatment usage, including how different insomnia treatments are prescribed
- Attitudes toward a blinded insomnia treatment profile
- Relationship with patients

To be eligible for participation in this survey, healthcare professionals (HCPs) needed to have been in their current role for at least 3 years, to spend at least 70% of their time seeing patients, and to treat at least 15 adult patients with insomnia per month. Participating physicians were recruited from a professional HCP panel for market research, for which they had previously registered and provided consent to be contacted for market research studies. Participants who met the eligibility criteria and completed the questionnaire received compensation for their time.

### 2.2. Secondary research on longitudinal prescriptions relating to insomnia

To evaluate whether the HCPs’ estimate of BZD and ZD use was aligned with prescribing data, longitudinal patient prescription activity was analyzed for European countries of interest with relevant datasets available (France, Germany, and the United Kingdom). In each of these countries, data for the top 5–6 molecules was acquired.

The analyzes of longitudinal prescriptions relating to insomnia were originally performed in March 2021. For France and Germany, IQVIA Longitudinal Prescription Data (LRx) datasets (IQVIA)^2^ of retail pharmacy prescriptions were screened for patient prescriptions relating to sleeping disorders. For the United Kingdom, the IQVIA Longitudinal Patient Dataset (LPD) containing information from electronic medical records of GP-treated patients was screened for prescriptions relating to sleeping disorders. The LRx and LPD datasets allowed a look-back period of at least 12 months. All analyzed data were anonymized (no patient identifiers were present) and GDPR compliant.

### 2.3. Secondary research on volume per class for medicines prescribed in insomnia

In this study, total volume of sales with an indication for insomnia [specifically, prescription of non-barbiturate hypnotics/sedatives in the European Pharmaceutical Marketing Research Association’s N5B1 anatomical classification for the treatment of insomnia (35)] over the previous 12 months was calculated for the five European countries of interest using data from the IQVIA MIDAS® platform (IQVIA, see text footnote 2). The N5B1 classification includes BZDs which are used mainly for insomnia, ZDs and melatonin receptor agonists. BZDs used for other indications and sedating antidepressants are categorized within the N5C (Antidepressants and Anxiolytics) classification, so were not included in our analyzes. The MIDAS® platform provides an estimate of national-level sales to retail and/or hospital pharmacists from data collected at a local level. To compare data for medicines having different dosing regimens, standard units were defined to represent the smallest daily unit of consumption for each drug of interest, e.g., one tablet. Results for each drug class are presented as the proportion of standard units for that drug per million standard units of N5B1 medicines sold.

## 3. Results

### 3.1. Primary research among healthcare professionals working in insomnia

Between April 2021 and May 2021, this study enrolled 602 HCPs from five representative European countries (France, Germany, Italy, Spain, and the United Kingdom; Table 2). GPs made up the highest proportion of participants (*n* = 361; 60.0%), followed by psychiatrists (*n* = 161; 26.7%), neurologists (*n* = 41; 6.8%), and sleep specialists (*n* = 39; 6.5%). These proportions were consistent across the five countries surveyed.

**Table 2:** Healthcare professionals participating in online survey on management of insomnia.

	Total	General practitioners	Sleep specialists	Neurologists	Psychiatrists
United Kingdom	120	72	6	9	33
Spain	120	72	7	9	32
Italy	120	72	8	10	30
Germany	121	73	7	7	34
France	121	72	11	6	32
Total	602	361	39	41	161

**Figure 1:**
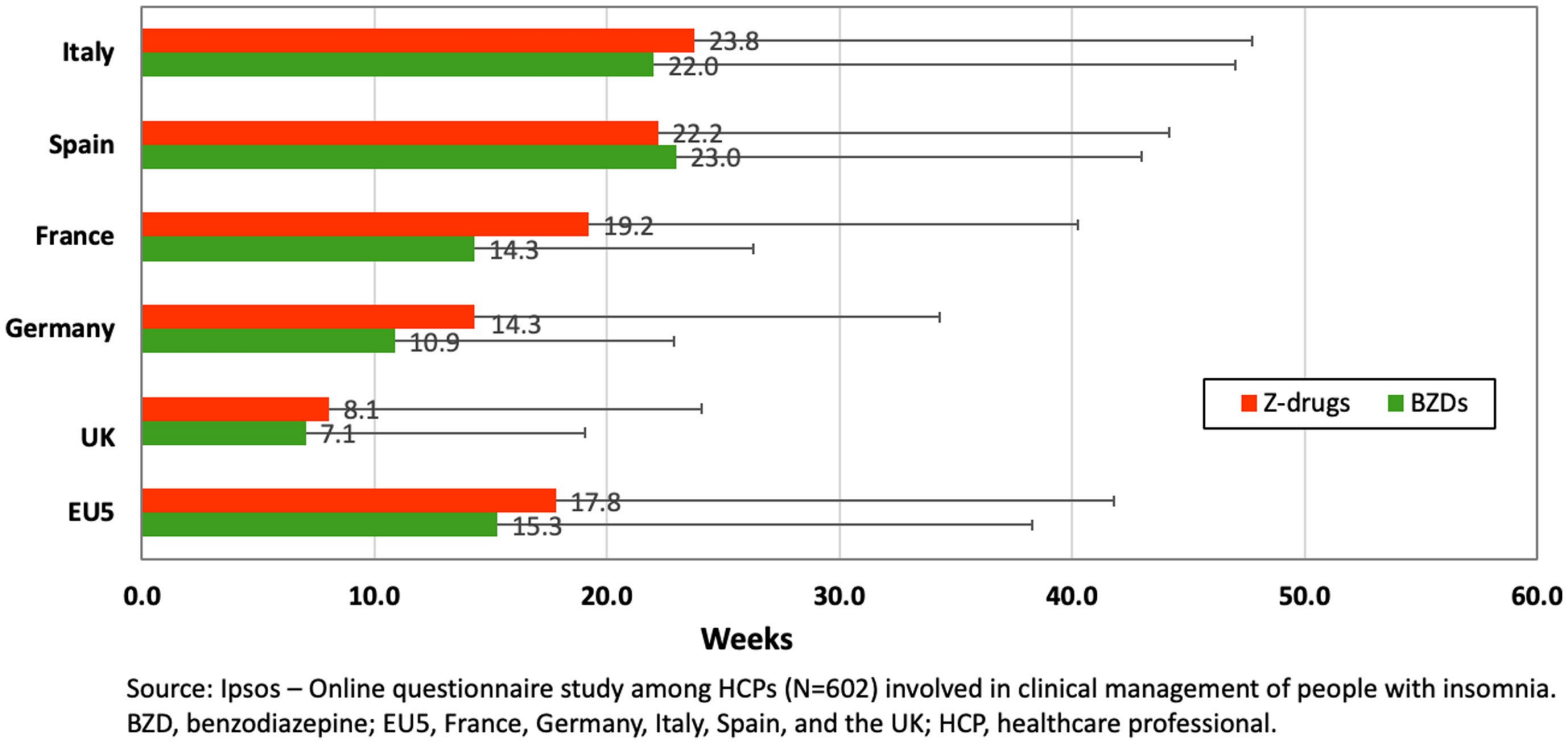
HCPs’ estimated duration of use across drug classes in insomnia (mean + standard deviation).

When asked to estimate their typical duration of use of BZDs and ZDs in patients with insomnia, across the five countries, the average was 15.3 [standard deviation (SD): 23.0] weeks for BZDs [ranging from 7.1 (SD: 12.0) weeks in the United Kingdom to 23.0 (SD:20.3) weeks in Spain] and 17.8 (SD: 23.9) weeks for ZDs [ranging from 8.1 (SD: 16.3) weeks in the United Kingdom to 23.8 (SD:24.3) weeks in Italy] (Figure 1).

Across the countries surveyed, HCPs acknowledged that their use of BZDs and ZDs typically extends beyond the limit of ≤4 weeks recommended for these agents (27) and the drug labels. As these survey results are based on the HCPs’ recollection, the average duration of use may be subject to bias. For example, results may be impacted by recall bias (e.g., not remembering previous events or experiences accurately or omitting details) or social desirability bias (e.g., underestimating the actual duration of treatment, if respondents perceived that a shorter duration of use was preferred by their peers) (36).

### 3.2. Secondary research on longitudinal prescriptions relating to insomnia

**Figure 2:**
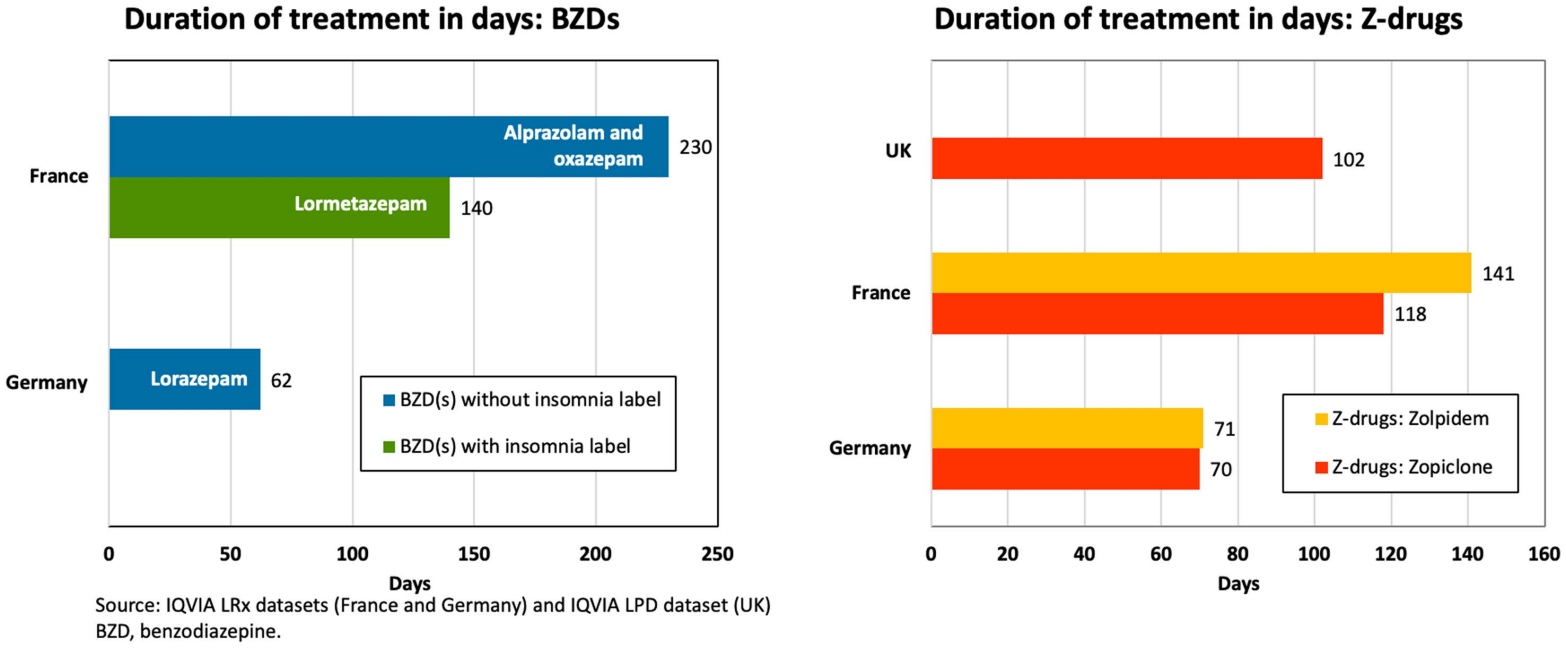
Longitudinal prescriptions in insomnia: mean duration of treatment.

The longitudinal prescription data showed that the average duration of treatment with BZDs prescribed for insomnia ranged from 62 days (8.9 weeks) for lorazepam in Germany to 230 days (32.9 weeks) for alprazolam and oxazepam in France (Figure 2). There were insufficient data for BZD prescriptions for insomnia in the United Kingdom LPD dataset to calculate the average duration of BZD use in this country.

Across the three countries examined, the average duration of treatment with ZDs ranged from 70 days (10 weeks) to 141 days (20.1 weeks) (Figure 2). In France and Germany, patients with insomnia were mainly treated with either zolpidem or zopiclone; whereas, in the United Kingdom, only zopiclone was prescribed.

Despite prescribing data not being available for all five of the countries of interest, the results that were available support the HCP questionnaire survey findings, demonstrating that the duration of BZD and ZDs prescriptions in Europe extend beyond the recommended limit of ≤4 weeks for these agents.

### 3.3. Secondary research on volume per class for medicines prescribed in insomnia

**Figure 3:**
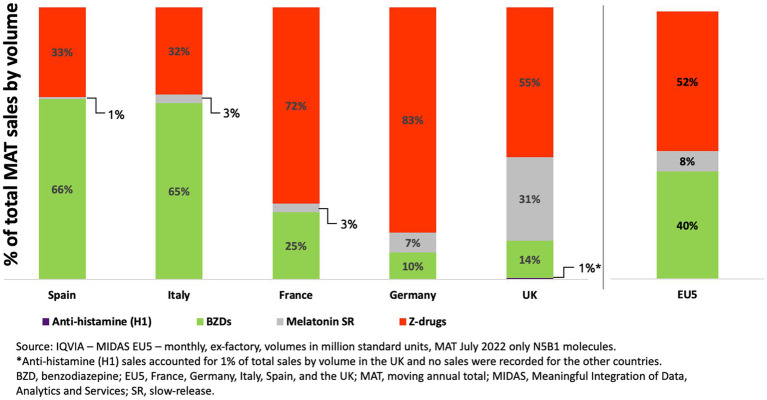
Sales by volume for classes of N5B1 medicines prescribed in insomnia.

Analyzes performed in July 2022 showed that, across the five countries examined, BZDs and ZDs accounted for 92% of the total volume of prescribed medicines indicated for insomnia (Figure 3). The sales by volume for BZDs were highest in Italy and Spain, where they made up 66.3 and 64.8% of the total, respectively. In contrast, in Germany and the United Kingdom, the sales by volume for BZDs were much lower, at 14 and 10% of the total, respectively. The sales by volume for ZDs ranged from 32% of the total in Italy to 83% of the total in Germany. Across the five countries, sales by volume of long-acting formulations of melatonin were lower than that for BZDs and ZDs, with the exception of the United Kingdom, where melatonin accounted for 31% of sales versus 14 and 55% for BZDs and ZDs, respectively. Long-acting melatonin is licensed for use in adults aged >55 years old for up to 13 weeks as a treatment for primary insomnia (37), however the current European guideline does not recommend melatonin for the treatment of insomnia because of low efficacy (27).

These data show that BZDs contribute a high proportion of total sales by volume for N5B1 medicines prescribed in insomnia (on average, 40% across the five countries examined). It is also important to consider that there may be additional prescribing of BZDs that do not have a licensed indication for use in insomnia (e.g., alprazolam, lorazepam, and oxazepam). Thus, even in countries with apparently lower BZD sales by volume (Germany and the United Kingdom), there may be additional usage of BZDs that is not captured in the data shown.

## 4. Discussion

The results from this series of pilot market research studies demonstrate that BZDs are widely used across European countries in the management of insomnia and the duration of treatment is longer than recommended. To determine whether published data align with these findings, we examined relevant areas of the literature (it should be noted that most published reports on BZD duration of use are not specific for use in insomnia, but are believed to include a proportion of patients using BZDs for this indication).

Both acute and chronic insomnia symptoms have a high prevalence in the general population, partly due to insomnia disorder often being comorbid with psychological or somatic disorders (38). The 3P model for insomnia etiology suggests that predisposing, precipitating, and perpetuating factors influence the onset and maintenance of chronic insomnia (39). Predisposing factors exert influence throughout the entire course of the disorder making some individuals more vulnerable than others. Precipitating factors (such as stress) may fuel early insomnia, however their impact may diminish over time, whereas perpetuating factors, such as learned negative associations, may keep the individual over the insomnia threshold, resulting in chronic insomnia. In cases where insomnia symptoms are secondary to another condition, insomnia symptoms may persist after successful treatment of the main disorder due to perpetuating factors, so it is important for the physician to monitor how the patient is sleeping (38) and to continue to seek approaches to identify predisposing and perpetuating factors, even after the natural course of the precipitating factor(s) has subsided (40).

In Europe, patients suffering from chronic insomnia typically visit their GPs after trying solutions such as over-the-counter sleeping aids, lifestyle changes (e.g., reducing caffeine intake, sleep hygiene), and, in some cases, meditation, yoga, etc. Due to the wide impact of chronic insomnia on multiple aspects of health and wellbeing and the heterogeneous population that suffers from this disorder, the management of chronic insomnia would ideally involve a multidisciplinary collaboration among HCPs such as GPs, psychiatrists, neurologists, and psychologists. However, in clinical practice, GPs often attempt to solve chronic insomnia with limited resources, e.g., lack of time for patient consultations, limited up-to-date knowledge about the condition, limited awareness and/or availability of CBTi, and lack of approved pharmacotherapy for chronic insomnia. As a consequence, if improvements in sleep hygiene (and CBTi, if tried) fail, GPs (and also specialists) may resort to using medications such as BZDs and ZDs for longer than recommended to treat chronic insomnia. Psychological and physical dependence on BZDs, which can develop within a few weeks of regular or repeated use (41) may also contribute to a longer duration of use.

Current pharmacological treatments may create a paradoxical situation: on one hand, such drugs should not be used long term, and on the other hand, they are being used to manage a chronic disease, which, by its own definition, lasts at least 3 months (24, 25). A second challenge is that although substance abuse/dependence are recognized as comorbidities in chronic insomnia (Table 3) (20), BZDs that are used in treating insomnia carry the risk of tolerance and dependency (42). Furthermore, although the use of BZDs and ZDs is supported by moderate-quality evidence for the short-term treatment (≤4 weeks) of insomnia, with the exception of eszopiclone (43), the long-term efficacy of these drugs is not proven (33).

**Table 3:** Major comorbidities of insomnia.

Psychiatric	Medical	Neurological	Substance use/dependence
Depressive disorders Bipolar disorder Generalized anxiety disorder Panic disorder Post-traumatic stress disorder Schizophrenia	Chronic obstructive pulmonary disorder Chronic kidney disease Diabetes mellitus HIV infection Malignancy Rheumatic disorders Chronic pain Sleep apnea	Neurodegenerative disease Fatal familial insomnia Cerebrovascular disease Multiple sclerosis Restless legs syndrome	Alcohol Nicotine Caffeine Marijuana Opioids Designer drugs Cocaine Amphetamine

Adapted from Riemann et al. (27). HIV, human immunodeficiency virus.

### 4.1. Scale of the problem: prevalence of long-term BZD use

In our study, the HCP-estimated duration of use for BZDs was longest in Italy and Spain at 22 and 23 weeks, respectively, and shortest in Germany and the United Kingdom (at 10.9 and 7.1 weeks, respectively). Our pilot study methodology was not structured to determine factors which might explain differences in the pattern of BZD use between the countries surveyed. However, a recent global analysis of BZD and ZD sales data found that increased consumption of these drugs in countries was statistically associated (*p* < 0.05) with several factors including increased prevalence of anxiety, self-harm and neurological disorders (44). Other factors, such as differences in prescribers’ attitudes to the risks and benefits of long-term use of BZDs between countries may also be involved.

Globally, the prevalence of long-term BZD use is estimated at approximately 2.0–7.5% in the general population, with estimates for long-term use among BZD users ranging from 25 to 76% (45, 46). Focusing on European countries, the results appear to echo the global pattern. High use of BZDs can be seen across Europe, with variations between individual countries. France shows a particularly high use of BZDs compared with other European countries (47), as does Italy, where, according to a 2018 report of the Italian National Observatory on the Use of Medicines (Osservatorio Nazionale sull’Impiego dei Medicinali), BZDs are among the most purchased medications paid for in full by patients alongside ZDs (48). Further European data also establish that the duration of BZD use is often higher than guideline recommendations.

High proportions of people taking BZDs do so on a long-term basis, with 95.8% of all BZD prescriptions (in any indication, including insomnia) identified as being used off-label for longer than 3 months in Spain (49). Use of BZDs beyond 12 months is also evident elsewhere in Europe, with ~20% of all BZD prescriptions for any indication in Germany (50) and an estimated 35% of prescriptions for any indication in the United Kingdom (51) being of this duration. A median duration of BZD use (for any indication) of 7 months was recorded in one study in France (52), while in Italy, 64.2% of patients with insomnia had been taking hypnotics/sedatives for >3 years (53). Data from the Berlin Aging Study show that the mean period of BZD use for any indication was 7.6 years; 33.8% of patients in this study were treated with BZDs for 1–5 years, while 40.3% were treated with BZDs for >5 years (54, 55). In addition, COVID-19 lockdown confinements resulted in an increase in the use of hypnotics, including BZDs over the period between March 2020 and April 2021, with an additional 1.4 million deliveries of hypnotics compared to expected being recorded in France (56).

BZD misuse has also been observed among HCPs, who are at a high risk of sleep disorders linked to work-related stress. HCPs who are dependent on BZDs experience difficulties at work due to cognitive impairment, but they are usually ashamed to seek medical help and worried about the potential legal implications of their dependence (57, 58).

These examples from the literature further support the market research evidence that BZDs are used for longer than their indications including in chronic insomnia.

### 4.2. Challenges associated with long-term use of BZDs for the treatment of chronic insomnia

#### 4.2.1. Development of dependence and tolerance can arise over time

While the risk–benefit ratio for BZDs remains positive in most patients over the indicated use of 2–4 weeks, this ratio is not stablished beyond that duration because of the difficulty of predicting the risk of dependence (42). There is an increased risk of dependence with long-term use; an estimated 20–50% of patients are believed to experience some sort of withdrawal when trying to discontinue BZDs (59), and 3–4% of them display clear signs of dependence (42, 60, 61). One study in France found that nearly half (49.8%) of adults aged 18–64 years old who were chronic users of BZDs (≥3 months and taking a BZD for any indication, including insomnia) were suffering from physical and psychological dependence (62).

Tolerance develops within a short time frame of 3–14 days of continuous BZD use, with effects of BZDs often wearing off after a few weeks and the potential of rebound insomnia with drug cessation (45, 63, 64). This high potential of tolerance, combined with the very low toxicity of BZDs, can induce consumption at extreme doses in an effort to maintain the hypnotic effect, whereas the effects of high-dose BZD withdrawal syndrome are very poorly tolerated and risky for the patient’s health (45, 65), so it can be difficult to wean patients from using these agents.

#### 4.2.2. Pharmacology of BZDs and ZDs needs to be considered

BZDs and ZDs work by potentiating gamma-aminobutyric acid (GABA) activity (66), the principal inhibitory neurotransmitter in the human brain (67). While BZDs bind to the alpha-1, −2, −3, and − 5 GABA receptor subunits, ZDs selectively bind to the alpha-1 receptor subunit (66). Although the primary sites of action of BZDs are the GABA receptor subunits, they also indirectly enhance dopamine release in the nucleus accumbens (68). This increase in dopamine concentration is responsible for the addictive properties of BZDs and ZDs (69, 70).

In general, BZDs can offer anxiolytic (anxiety-reducing) or hypnotic (sleep-inducing) effects, with overlap between the two properties (71). BZDs can be categorized as short-acting [half-life (T_1/2_) <24 h] and long-acting treatment (T_1/2_ > 24 h) (72). BZDs available for use in insomnia include triazolam (T_1/2_: 1.5–5.5 h) (73), temazepam (T_1/2_: 8–15 h) (74), lormetazepam (T_1/2_: 11 h) (75), loprazolam (T_1/2_: 12.8 h) (76), nitrazepam (T_1/2_: 24–40 h) (77), flunitrazepam (T_1/2_: 18–26 h) (78), flurazepam (T_1/2_: 2.3–3.5 h; T_1/2_ of N1-des-alkyl-flurazepam: 19–133 h) (79), clonazepam (T_1/2_: 20–60 h) (80), and nordazepam (T_1/2_: 36–200 h) (81). Compared with BZDs, ZDs show a shorter duration of action and half-life (zopiclone, T_1/2_: 4–5 h; zolpidem, T_1/2_: 2.5 h; zaleplon, T_1/2_: ~1 h). Although their fast clearance helps minimize undesirable residual effects, too short a half-life can be a problem if patients require both sleep initiation and sleep maintenance therapy (82).

Short-acting hypnotics are preferable in patients with sleep onset insomnia, when sedation the following day is undesirable, or when prescribing for elderly patients. Long-acting hypnotics are indicated in patients with poor sleep maintenance (e.g., early morning waking) that causes daytime effects, when an anxiolytic effect is needed during the day, or when sedation the following day is acceptable (83). The risk-benefits of long- versus short-acting BZDs need to be considered on an individualized basis. For example, for short-acting BZDs relative to long-acting BZDs, withdrawal phenomena are more common (83, 84), and among people aged 65 years and older, there is a higher risk of developing dementia {adjusted odds ratios of 1.98 [95% confidence interval (CI): 1.89–2.07] and 1.47 [95% CI: 1.37–1.58] versus non-users of BZDs respectively} (85). In comparison, a meta-analysis found that long-acting BZDs are associated with a significantly higher risk for hip fractures than short-acting BZDs [relative risk: 1.31 (95% CI: 1.18–1.45) versus 1.15 (95% CI: 1.08–1.22), *p* < 0.0001, relative to non-BZD users, respectively] (86). A study in residential aged care facilities in Australia suggested that residents who used long-acting BZDs on a regular basis had higher night-time sleep quality versus those using short-acting BZDs taken on an as-needed basis (87).

Although ZDs have a shorter duration of action and more rapid clearance than BZDs, they can impair nocturnal and next-day psychomotor performance, including cognition, behavior, psychomotor performance, and driving ability (82, 88). Higher dose ZD use in people with dementia is associated with increased fracture and stroke risks, similar or greater to that for higher dose BZDs (89). In the United States, the FDA has required that all ZDs carry a Boxed Warning highlighting the risk of complex sleep behaviors (e.g., sleep-walking, falls and sleep-driving) which can result in serious injuries, including death (90).

#### 4.2.3. Cognitive and central nervous system effects can impact patients’ lives

The negative effect of long-term use of BZDs on cognitive function has long been known, with meta-analysis data having reported moderate-to-large effects for all cognitive domains in long-term BZD users, including increased risk of motor vehicle collisions compared with non-BZD users (91, 92). An increased risk of development of dementia among BZD users has also been found in meta-analyzes (93, 94). This increase was observed for users of both long- and short-acting BZDs relative to non-users of BZDs (93). Studies in mice suggest that long-term but not short-term use of BZD impairs motoric function and upregulates neurotoxic amyloid β42 in part through suppression of neuroprotective translocator protein in the cerebellum, suggesting a potential mechanism for movement deficit and increased risk of falls seen in chronic BZD users (95).

Long-term use of BZDs is also correlated with further insomnia, anxiety, drug dependence, and depression (96). This may be a factor involved in the pattern of long-term use of BZDs without a label for insomnia that we observed, i.e., lorazepam, alprazolam and oxazepam (Figure 2). It is known that insomnia affects around half of individuals with anxiety (97), so by using these drugs, physicians may be attempting to address both insomnia and anxiety, despite their not being licensed for insomnia, and the products being licensed for short-term relief only in the treatment of anxiety due to concerns about tolerance and dependency (98). Physicians may keep using these BZDs for longer than recommended due to the lack of approved pharmacotherapy for chronic insomnia and/or the difficulties associated with BZD withdrawal syndrome mentioned previously.

High-dose BZD-dependent patients have been reported to have reduced quality of life directly associated with BZD intake compared with non-BZD users, both from a physical and emotional position, reduced social functioning, and high levels of psychological distress (99, 100).

Further evidence also warns against the long-term use of BZDs due to associations with long-term unfavorable effects, such as oversedation, depression, and impairment of the immune system (101). It has also been reported that, in contrast to short-term use at low therapeutic doses (which normalizes sleep microstructure), long-term use of high doses of a BZD for chronic insomnia induces a severe disruption of sleep microstructure (102).

#### 4.2.4. Recommendations and contraindications in the product label may not be followed

Failure to adhere to recommendations and contraindications in the product label can occur in patients at risk of drug–drug interactions, patients with certain pre-existing medical conditions, or patients who are at a high risk for treatment-related adverse events (74, 75). For instance, high-dose use of BZDs and ZDs were found to be associated with attention deficit hyperactivity disorder (ADHD) (103), and use of high dose levels of long-acting BZDs, short-acting BZDs and Z-drugs were all found to significantly increase the risk of fall-related injuries requiring hospitalization in people aged 65 years and older (104). Due to the increased risk of falls, care is recommended when using BZDs in elderly subjects (27). As ADHD is often associated with sleep disturbance, caution should also be taken when prescribing BZDs or ZDs in people with ADHD (105). Furthermore, BZDs may lower the threshold for suicidal behavior in patients with depression (71).

Caution is also needed for patients who are using other medications that may produce additive effects when co-administered with BZDs. This includes drugs/substances that produce depression of the central nervous system (CNS), such as alcohol, antipsychotics, anxiolytics, antidepressants, narcotic analgesics, sedative antihistamines, and anticonvulsants (74, 75). As CNS suppressants, BZDs and ZDs can also act as respiratory depressants by suppressing one or more steps in respiration and patency (106). Indeed, long-term BZD use may cause complete obstructive sleep apnea in heavy snorers or short repetitive central sleep apnea in patients with recent myocardial infarction (107). In older patients (aged ≥66 years) with chronic obstructive pulmonary disorder, initiation of treatment with a BZD increases risk for outpatient respiratory exacerbations (108). Additionally, care should be taken for patients taking muscle relaxants, as the overall muscle-relaxing effect of BZDs may accumulate and increase the risk of falling in elderly patients and those on higher doses compared with those not on these medications (74, 75).

Before prescribing BZDs and ZDs in any patient with chronic insomnia, the risk of substance abuse should be assessed, as these drugs should be avoided in individuals with a history of drug or alcohol abuse (20, 77).

#### 4.2.5. Patients may misuse BZDs without understanding the risks

Ineffective communication of the health risks associated with long-term use and gaps in communication between patients and HCPs can contribute to the problem of misuse (109). Patients may be unwilling to listen to their HCP’s advice on the health risks of their medication and instead perceive BZDs as having a subjective positive risk–benefit ratio. If discontinuation is discussed by their HCP, they may seek to switch to another HCP who is more willing to prescribe BZDs (110).

Patients taking BZDs may have concerns that alternative therapies are less effective for treating chronic insomnia, and this commonly combines with apprehension of drug cessation without professional support and a fear of recurring symptoms upon discontinuation (109, 110).

Moving forward, it is fundamental that patients with chronic insomnia are investigated for the cause of their symptoms (predisposing, precipitating, and perpetuating factors), something that is often neglected because of a lack of resources for the management of long-term medication use, especially in older adults (109). Patients should also be evaluated for risk factors that may affect their BZD use, including likelihood of abuse and dependence, before prescription of any drug (111).

### 4.3. Recent links between BZD use and risk of opioid use

The link between use of BZDs and increased risk for future opioid abuse is an emerging area that warrants further research, especially as patients with insomnia are at risk of substance use/dependence (including opioids) as a comorbidity (27). While overall opioid consumption per person and opioid-related mortality rates in Europe remain lower than in the United States (which has faced an opioid crisis over the past 20 years) (112–114), the use of prescription opioids is growing, with 1.3 opioid-related deaths per 100,000 inhabitants in the EU in 2017 (112). Although BZDs are not indicated for pain control and are an ineffective pain medication, they are frequently taken by patients for pain, and taken concomitantly with opioids (115). Efforts to control the growing opioid crisis should include focusing on the use of BZDs as a risk factor.

Use of BZDs is known to increase the risk of misuse of prescribed opioids and the likelihood of future chronic use of opioids (113, 116–118). Some people may use BZDs alongside opioid-based drugs to potentiate or extend their effects, and this may serve as a predictor for more severe polysubstance use problems (119), as concomitant use of BZDs and opioids may result in sedation, respiratory depression, coma, or fatal intoxication (113, 116–118, 120, 121). An Italian study identified that over 20% of individuals who were high-dose misusers of BZDs were also polydrug (17.2%) or former polydrug users (4.3%) of heroin, cocaine, and alcohol (122).

Individuals who have previously used BZDs have more than double the risk of misusing opioids compared with those who have not used BZDs (116). They also have up to nearly a 15-fold greater risk of drug-related death than individuals not prescribed either drug (113). Having a history of BZD use is a risk factor for both “opioid shopping” behavior (where patients obtain overlapping opioid prescriptions from different prescribers that are then filled in multiple pharmacies) and opioid abuse (123). Studies using population health survey findings crosslinked to the Norwegian prescription database show that a history of BZD use raises the chance of being prescribed opioids up to 20 years later in life among those who also used alcohol. Follow-up analyzes also demonstrate that use of BZDs predicts repeated use of opioids 4–7 years later, and that the use of BZDs is a strong predictor of later opioid use (124, 125).

Other European data show that despite warnings in the BZD product labels against using these agents together, concomitant use of BZDs with opioids does occur. In Spain, a study of real-world patterns of opioid use in the Valencia region (~5 million inhabitants) found that almost 25% of people initiating treatment with opioids between 2012 and 2018 had overlapping use of BZDs (126). Similarly, a population-based registry study of BZD use in the Castile and León region (~2.4 million inhabitants) in 2016 reported that almost 25% of individuals using a BZD daily also used opioids (127). Misuse of BZDs and illicit opioids has also been shown to be a problem for hospitals across Europe. In Italy, a top cause of access to emergency medical care was misuse of BZDs in individuals addicted to heroin (128). Italian data analyzing hospital admissions to the addiction unit from 2003 to 2010 also highlighted that 32% of admissions were for BZD abuse, with 25.2% of this patient subgroup showing addiction to BZDs and illicit drugs, including opioids (129). Previous BZD use can also increase the likelihood of chronic opioid use after surgery, with one Finnish study reporting that prior BZD use doubled the probability of chronic opioid use at 12 months after elective orthopedic surgery (130, 131).

## 5. Conclusion

Insomnia is associated with numerous negative health effects (27) and chronic insomnia remains a major burden for individuals and healthcare systems globally (111). The lack of training and availability of clinical staff limit the use of CBTi (33). BZDs and ZDs have a place in the treatment pathway for chronic insomnia, however, there is also an unmet need as these and other current pharmacological treatments are not licensed for use beyond 4 weeks and so are not aligned with the chronicity of the disease (i.e., symptoms lasting >3 months). The lack of wider CBTi availability and licensed alternative pharmacotherapy options (especially for chronic insomnia) appears to contribute to both undertreatment of chronic insomnia (14) and widespread use of BZDs and ZDs beyond their recommended duration of treatment.

In patients who have been using BZDs for longer than recommended, stopping or reducing the use of BZDs may be difficult due to the withdrawal symptoms. However, long-term use of high doses of BZDs (e.g., in individuals where the dosage was increased over time due to tolerance developing) induces a severe disruption of sleep microstructure (102), so any initial efficacy in improving symptoms diminishes over time. Patients with symptoms of BZD dependence and addictions are not coming forward to HCPs and clinics, so it is challenging for doctors to recognize these patients. To date, there is little research on characterization of high-dose BZD users in chronic insomnia, so the next step is to ensure that clinicians can identify patients with risk factors for escalation of BZD use.

Guidance is available for deprescribing and withdrawing of BZDs (132, 133), however not all HCPs may be familiar with or use this. Evidence has shown that in both short- and long-term scenarios, deprescribing BZDs by gradual tapering in combination with non-pharmacological support is more likely to succeed than gradual tapering alone (134). A multicenter, randomized interventional study in Belgium recently looked at strategies to support BZD discontinuation in primary care (135). This study found that the use of blended care, which combines the use of an interactive educational e-tool with face-to-face clinical consultations with the care provider, provides a complementary tool that enables the BZD discontinuation process to be tailored to the personal style of the GP and the needs of the patient.

In conclusion, there is a need to break the vicious cycle of chronic insomnia: chronic insomnia is a disorder that has substance use and dependence as major comorbidities (27), and yet it is treated with drugs such as BZDs and ZDs that carry a risk of tolerance, dependence, and misuse (for longer than recommended, at escalated doses over time, and/or in patients who are at risk of side effects). For patients with chronic insomnia, BZDs are likely to remain an appropriate option for short-term use; however, greater awareness of their limitations and risks when used for longer than recommended should encourage HCPs to stringently adhere to the treatment duration recommendations in clinical guidelines. Ultimately, treatment options suitable for long-term use in chronic insomnia are needed (136).

## Data availability statement

The original contributions presented in the study are included in the article/Supplementary material, further inquiries can be directed to the corresponding author.

## Author contributions

MS, IW, CL, FL, and GH: conceptualization. MS, IW, BC, CL, FL, and GH: methodology, investigation, data curation, writing–original outline and draft preparation, and writing–review and editing. All authors contributed to the article and approved the submitted version.

## Funding

This study received funding from Idorsia Pharmaceuticals Ltd. The funder had the following involvement with the study: design and commissioning of the market research studies conducted by Ipsos and IQVIA as described within this manuscript; funding of medical writing support provided by Patrick Foley and Olivia Taylor (NexGen Healthcare Communications, London, United Kingdom).

## Conflict of interest

BC and CL are employees of Idorsia Pharmaceuticals Ltd. IW was an employee of Idorsia Pharmaceuticals Ltd. at the time of preparation of the manuscript. FL has received payment for consultations, presentations, speaker bureaus, and educational events from Molteni Farmaceutici. GH has received payment for honoraria for lectures, presentations, speaker bureaus, manuscript writing or educational events from Bristol-Meyers Squibb, FEO, Gedeon Richter, Georg Thieme, Heel, Hexal, Hikma, Idorsia, Janssen-Cilag, Jazz, Lundbeck, Medfora, Medice, Medical Tribune, MedScape, MedTrix, Neuraxpharm, Pfizer, Prosomno, Recordati, Repha, Rovi, Sanofi-Aventis, and Schwabe. MS has received consultancy fees from Camurus.

The reviewer TW declared a shared affiliation with the author GH to the handling editor at the time of review.

## Publisher’s note

All claims expressed in this article are solely those of the authors and do not necessarily represent those of their affiliated organizations, or those of the publisher, the editors and the reviewers. Any product that may be evaluated in this article, or claim that may be made by its manufacturer, is not guaranteed or endorsed by the publisher.

## Supplementary material

The Supplementary material for this article can be found online at: https://www.frontiersin.org/articles/10.3389/fpsyt.2023.1212028/full#supplementary-material

## Footnotes

^1^ www.ipsos.com

^2^ www.iqvia.com
