# Supplementary material for: Long-term use of benzodiazepines in chronic insomnia: a European perspective
Source: Front Psychiatry. 2023 Aug 2;14:1212028. doi: 10.3389/fpsyt.2023.1212028 (PMC10433200; doi:10.3389/fpsyt.2023.1212028)
Supplement: Supplementary file 1 [file Data_Sheet_1.PDF]

Insomnia Quant HCPs

20-001550-01

QUESTIONNAIRE

Online survey
30 minutes

Version 3

Sample

	UK	Germany	France	Italy	Spain	Canada
**GPs**	72	72	72	72	72	72
**Specialists**	48	48	48	48	48	48
**Total**	**120**	**120**	**120**	**120**	**120**	**120**

Specialists target include a mix of: Neurologists, Psychiatrists & Sleep specialists. Recruit min 10 per group (min 30 for Psychiatrists).

Signed off for scripting / translation by:	
AD/D	
PM	

Screener
Summary

S1. Primary specialty	Must be a PCP, Sleep specialist, Neurologist or Psychiatrist
S2. Tenure	Must have been in current specialty/role between 3 and 35 years
S3. Time spent in direct patient care	Must spend at least 70% of time seeing patients
S4. Setting (hospital/office)	Hospital/office based by quota
S5. Setting (Type of practice – PCP/office)	Solo private, Private single-specialty, Private multi-specialty by quota
S6. Setting (Type of practice – hospital)	Community, university/teaching by quota
S7. Private vs. public patients	% of patients private/public by quota
S8. Insomnia pt. caseload	Must see at least 30 adult insomnia patients in a typical month
S9. Prescription behaviour	Must treat at least 15 insomnia patients with prescription therapy
S10. Insomnia research participation	Must not have participated in insomnia market research in the last 3 months
S11. Family employed by pharma	Must not personally or have a family member who is employed by a pharmaceutical company

Country. In which country do you live?

(Please select one answer only)

- ○ France
- ○ Germany
- ○ Spain
- ○ Italy
- ○ UK
- ○ Canada
- ○ None of these

[PN: SINGLE CODE. CLOSE IF 'NONE OF THESE' SELECTED. PRE-PUNCH FROM PANEL, DO NOT SHOW TO RESPONDENTS]

[Intro]

Intro. This survey is being conducted by **[A Third Party Agency],** an independent market research agency based in the UK, on behalf of a pharmaceutical company. We are carrying out research on **the perception and treatment of insomnia** and would like to ask you some questions on this topic. The survey will take approximately **30** minutes of your time. You will receive an honorarium for your participation.

With your consent, your information will only be collected and used for 'market research and analysis'**.** Any information you give will be treated in the strictest confidence and results will only be reported back on an aggregated basis.

As a member of the Market Research Society (MRS), **[Third Party Agency]** is bound by the MRS Code of Conduct and all applicable laws protecting your personal data and responses. The study is conducted in compliance with MRS/ ESOMAR/ EphMRA **[PN: UK ONLY -** / British Healthcare Business Intelligence Association**]** guides. You have the right to withdraw from the interview at any time. For more information about your rights and how data will be used, please see our privacy notice, it is available here **[PN: SCRIPTERS TO INSERT PRIVACY POLICY LINK].**

Consent. I confirm I do not need any consent from my employer, organization or professional association to participate in this research OR I have already obtained any consent needed.

(Please select one answer only)

- ○ Yes, I confirm I do not need consent or have already obtained it
- ○ No, I do not have consent or have not already obtained it

[PN: SHOW ONLY IN DE & IT. SINGLE CODE. CLOSE IF 'NO' SELECTED]

[Screener]

S1. Which of the following best describes your primary specialty?

1. Neurologist
2. Psychiatrist
3. Sleep specialist
4. Primary care physician/General practitioner
5. Other (please specify) **[PN: TERMINATE]**

[PN: SINGLE CODE – RECRUIT BASED ON QUOTA]

S2. Approximately how many **years** have you been qualified in your current specialty / role?

________years qualified

[PN: OPEN NUMERIC RANGE 0 TO 70]
[PN: TERMINATE IF LESS THAN 3 OR MORE THAN 35]

S3. What percentage of your professional time do you spend in clinical practice treating patients (as opposed to time spent in clinical research, teaching or administrative duties)?

_________% of professional time seeing patients

[PN: OPEN NUMERIC % - RANGE 0% TO 100%]
[PN: TERMINATE IF LESS THAN 70%]

ASK ALL EXCEPT UK

S4. In which setting do you spend the majority of your time?

1. Hospital based
2. Office based

[PN: SINGLE CODE – RECRUIT BASED ON QUOTA]
QUOTA: 60% OF NEUROS SHOULD BE OFFICE-BASED

ITALY: GPs, Sleep specialists and psychs
50% Hospital Base
50% Office Based

FRANCE
GPs and Psychs: 100% Office Based
Sleep specialists: 20% Office Based
80% Hospital Based

GERMANY: GPs, Sleep specialists and psychs
50% Hospital based
50% Office based

CANADA: GPs, Sleep specialists and psychs
65% Hospital Based
35% Office Based

SPAIN: GPs, Sleep specialists and psychs
50% Hospital based
50% Office based

ASK PCPs and OFFICE BASED DE PHYSICIANS
S5. Which of the following best describes your primary practice setting?

1. Solo private practice
2. Private single-specialty group practice

3. Private multi-specialty group practice
4. Other (please specify)

[PN: SINGLE CODE – SOFT QUOTA RECRUIT MIX]

ASK ALL HOSPITAL BASED SPECIALISTS
S6. Which of the following best describes your primary practice setting?

1. Community hospital practice
2. University/ Teaching hospital practice
3. Other (please specify)

[PN: SINGLE CODE – RECRUIT BASED ON QUOTA]
QUOTA:
UK:
50% Community
50% University

ITALY:
Recruit even mix

FRANCE
Recruit even mix

GERMANY:
65% Teaching Hospitals
35% Community hospitals

CANADA:
40% Community
25% University
35% Other

SPAIN:
Recruit even mix

S7. What percentage of your professional time do you spend treating patients with private health insurance vs publicly insured patients?

1. ___% of professional time spent treating privately insured patients
2. ___% of professional time spent treating publicly insured patients

[PN: OPEN NUMERIC RANGE 0-100% - TOTAL MUST EQUAL 100%]
[PN: RECRUIT TO QUOTA]
QUOTA: EX- UK AND FR: Min 50% to spend at least 30% of their time treating privately insured patients
UK AND FR: Min 30% to spend at least 30% of their time treating privately insured patients

S8a. How many patients are currently under your care and for whom you make treatment decisions?

________patients

[PN: OPEN NUMERIC]

S8. How many **adult** patients with **insomnia** do you **personally manage for their insomnia** in a typical **month**?

________adult patients with insomnia

[PN: OPEN NUMERIC]
[TERMINATE IF LESS THAN 30]

S9. Of the **[INSERT FROM S8]** adult patients you treat with insomnia in a typical month, for how many do **you personally prescribe** the following options?

1. Number prescribed prescription therapy **[PN: OPEN NUMERIC]**
2. Number prescribed or recommended an OTC therapy **[PN: OPEN NUMERIC]**
3. Number recommended non-pharmacological interventions ONLY **[PN: OPEN NUMERIC]**

[PN: TERMINATE IF LESS THAN 15 PATIENTS ON PRESCRIPTION THERAPY]

S10. Have you participated in market research on the subject of **insomnia** in the past three months?

(Please select one answer only)

- ○ Yes, I have participated in market research on insomnia in the past three months
- ○ No, I have not participated in market research on insomnia in the past three months

[PN: SINGLE CODE. CLOSE IF 'YES' SELECTED]

S11. Are you or any family member employed by a pharmaceutical company in any capacity (other than within the context of clinical trials, advisory boards etc.)?

(Please select one answer only)

- ○ Yes
- ○ No

[PN: SINGLE CODE. CLOSE IF 'YES' SELECTED]

Section E: Treatment usage & attitudes

[CONSTRUCTS FOR INTERNAL REFERENCE ONLY]

Current treatment evaluation: usage, satisfaction, preference, perceived efficacy and safety, perception of benzos vs. Z medications vs. antihistamines, melatonin, antidepressants etc.; reasons to switch treatment; reasons to stop treatment
Current prescribing habits: tendency to use a particular medication or medication class as the first line choice/ for certain patient groups
Unmet needs: current perceived unmet needs for insomnia treatments (overall and for specific patient groups)
View on access hurdles: current access barriers to overcome; willingness to fight access hurdles for a new effective insomnia treatment
Willingness to adopt new therapies for insomnia: likelihood to be early adopters of new therapies vs. waiting for others to use first; willingness to try innovative / targeted therapies for insomnia; current perception of insomnia treatment development (level of innovation in the field/ attention from pharma); awareness of new MOA; excitement in trying new options

[CONSTRUCTS END]

E1. Thinking about your current insomnia patients, please select your **first and second choice** when it comes to prescription medication for treating their insomnia

[PN: RANKING]

1. Benzodiazepines (e.g. **[DE SHOW:** lorazepam, oxazepam, temazepam**] [FR SHOW:** alprazolam, lormetazepam, oxazepam**] [FOR IT SHOW:** alprazolam, lorazepam, lormetazepam**] [FOR ES SHOW:** alprazolam, flurazepam, lorazepam, **] [FOR UK SHOW:** alprazolam, lorazepam, temazepam**] [FOR CAN SHOW:** lorazepam, oxazepam, temazepam**]**)
2. Z-drugs (e.g. **[DE SHOW:** zolpidem, zopiclone**] [FR SHOW:** zolpidem, zopiclone**] [FOR IT SHOW:** zolpidem, zopiclone**] [FOR ES SHOW:** zolpidem, zopiclone**] [FOR UK SHOW:** zolpidem, zopicone**] [FOR CAN SHOW:** zolpidem, zopicone, eszopiclone, zaleplon**]**)
3. Melatonin (e.g. **[ALL EX-CAN SHOW:** Circadin**]**)
4. Antihistamines (e.g. **[DE SHOW:** promethazine**] [FR SHOW:** hydroxyzine**] [FOR IT SHOW:** hydroxyzine**] [FOR ES SHOW:** hydroxyzine**] [FOR UK SHOW:** hydroxyzine, promethazine**] [FOR CAN SHOW:** hydroxyzine**]**)
5. Sedative anti-depressants (e.g. **[DE SHOW:** mirtazapine, amitriptyline**] [FR SHOW:** mirtazapine, amitriptyline, mianserin, timipramine**] [FOR IT SHOW:** mirtazapine, amitriptyline, mianserin, trazodone**] [FOR ES SHOW:** mirtazapine, amitriptyline, mianserin, trazodone**] [FOR UK SHOW:** mirtazapine, amitriptyline**] [FOR CAN SHOW:** mirtazapine, amitriptyline, trazodone, doxepin**]**)
6. Antipsychotics
7. Anxiolytics
8. Other (please specify)

[PN: RANKING TOP 2]

E2. Now thinking about specific insomnia patient groups, please select your **first and second choice** when it comes to prescription medication for each of them.

[PN: GRID RANKING]
[PN: TOP, RESPONSES]

1. Patient with **chronic** insomnia (lasting longer than 3 months)
2. Patient with **acute** insomnia (lasting less than 3 months)
3. Active patients (working professional and/ or demanding lifestyle)
4. Non-active patients (retired, non-working and/ or slower lifestyle)
5. Patients with mental health conditions (e.g. depression, anxiety)
6. Patients with perceived higher risk of dependency
7. Elderly patients

[PN: LEFT, STATEMENTS]

1. Benzodiazepines
2. Z-drugs (zopiclone/ zolpidem)
3. Melatonin
4. Antihistamines
5. Sedative anti-depressants
6. Antipsychotics
7. Anxiolytics
8. Other (please specify)

[PN: SELECT 2 PER STATEMENT]

E3. Which insomnia treatment(s) do you associate the most with each of the following attributes?

(You can select more than one treatment for each attribute)

[PN: GRID]
[PN: TOP, RESPONSES]

1. Benzodiazepines
2. Z-drugs (zopiclone/ zolpidem)
3. Melatonin
4. Antihistamines
5. Sedative anti-depressants
6. Antipsychotics
7. Anxiolytics
8. Other (please specify)

[PN: LEFT, STATEMENTS]

1. Ability to improve speed of sleep onset
2. Ability to improve sleep maintenance
3. Ability to improve total sleep time
4. Ability to improve sleep **over time**

5. Ability to improve day time functioning
6. Does not cause morning grogginess
7. Overall tolerability
8. Safety for long term use
9. Does not cause dependency
10. Innovative mechanism of action
11. Patient's out of pocket cost
12. Patient acceptance
13. Flexible dosing
14. Absence of tolerance over time
15. High patient satisfaction
16. Low risk of drug-drug interactions with commonly taken medication
17. Negative impact on cognition
18. Recommended by guidelines
19. Other (please specify) **[PN: ANCHOR]**

[PN: MULTICODE PER STATEMENT, RANDOMISE STATEMENTS]

E4. To what extent do you agree or disagree with each of the following statement regarding switching prescription medication for your insomnia patients?

[PN: SCALE, USE CUSTOM SET-UP, STATEMENTS SHOWN ONE AT A TIME WITH SLIDER TO ANSWER]
[PN: TOP, RESPONSES]

1. Strongly disagree
2.
3.
4.
5.
6.
7. Strongly agree

[PN: LEFT, STATEMENTS]

1. I usually switch insomnia prescription medication within a short period of time if it is not helping the patient sleep well
2. I usually switch insomnia prescription medication within a short period of time if it is not improving the patient's next day functioning
3. If a patient is experiencing side effects, I ask the patient to stop taking the insomnia drug immediately
4. When an insomnia medication stops working, I always prefer switching the patient to another medication of the **same class** rather than a new class
5. I always try to titrate to the maximum dose of an insomnia drug before switching to any other drug

[PN: SINGLE CODE PER STATEMENT, RANDOMISE STATEMENTS]

E5. How long do you typically recommend your patients take an insomnia medication to observe the overall effect of the treatment before moving to another medication?

1. A few days
2. A week
3. A few weeks
4. A month
5. A few months
6. Half a year or longer

[PN: SINGLE CODE]

E6. For the majority of your insomnia patients, how long do they typically stay on prescription medication?

(Please answer in weeks OR months OR years)

____weeks

____months

____years

[PN: OPEN NUMERIC, ONLY ALLOWED TO ANSWER IN ONE ANSWER BOX]

E6b. Thinking now about different treatment classes, for the majority of your patients treated with each class, how long do they typically stay on each class of treatment?

[PN: ACROSS THE TOP]

1. Antihistamines
2. Benzodiazepines
3. Melatonin
4. Sedative anti-depressants
5. Z-drugs (zopiclone/ zolpidem)

[PN: DOWN THE SIDE]

1. Weeks
2. Months
3. Years

[PN: OPEN NUMERIC, ONLY ALLOWED TO ANSWER IN ONE ROW PER COLUMN]

E7. When thinking about the safety of insomnia medication, what do you typically focus on? Please rank your top 3.

1. Overall tolerability
2. Risk of complex sleep behaviours
3. Risk of dependency
4. Patient safety if they get up during the night (e.g. risk of falls)
5. Patient safety the next day (e.g. driving, operating machinery)
6. Long-term impact on cognition/ memory
7. Other (please specify) **[PN: ANCHOR]**

[PN: TOP 3, RANDOMISE STATEMENTS]

E8. What proportion of your insomnia patients on prescription medication report hangover effects/next day grogginess?

__% [PN: OPEN NUMERIC RANGE 0 TO 100]

E9. What is your typical action when a patient report hangover effects/ next day grogginess on their prescription medication?

[PN: LIST]

1. Lowering the dose
2. Lowering the frequency of dosing
3. Advising to take earlier in the evening
4. Stopping treatment
5. Switching to another prescription medication from a different class
6. Switching to another prescription medication within the same class
7. Helping patients to cope with effects
8. Not taking any actions
9. Other (please specify)

[PN: SINGLE CODE]

E10. To what extent do you agree or disagree with each of the following statements regarding discontinuing prescription medication for your insomnia patients?

[PN: SCALE, USE CUSTOM SET-UP, STATEMENTS SHOWN ONE AT A TIME WITH SLIDER TO ANSWER]
[PN: TOP, RESPONSES]

1. Strongly disagree
2.
3.
4.
5.
6.
7. Strongly agree

[PN: LEFT, STATEMENTS]

1. Once a patient has started sleeping well, I try to slowly get them off insomnia prescription drugs
2. Once an underlying mental health condition causing insomnia is resolved, I immediately reduce the dosing frequency of insomnia prescription medication
3. As long as the patient is not requesting repeat prescriptions too frequently, I am comfortable prescribing sleep medication over a longer period of time
4. I tell my patients to take their medication only for the period necessary but leave it to them to tell me when they feel ready to come off it
5. I always advise my patients to <u>not</u> take insomnia prescription drugs for longer periods
6. Even for chronic insomnia patients, I prefer <u>not</u> to prescribe insomnia prescription drugs for a longer period
7. Most of my patients prefer <u>not</u> to use prescription drugs for insomnia treatment

[PN: SINGLE CODE PER STATEMENT, RANDOMISE STATEMENTS]

E11. For each insomnia patient group below, please select the statements which apply regarding the prescription medication you have currently available.

[PN: DOWN, QUESTION OPTIONS]

1. Patients with **chronic** insomnia (lasting longer than 3 months)
1. Patients with **acute** insomnia (lasting less than 3 months)
2. Active patients (working professional and/ or demanding lifestyle)
3. Non-active patients (retired, non-working and/ or relaxed lifestyle)
4. Patients with mental health conditions (depression, anxiety)
5. Patients with perceived higher risk of dependency
6. Elderly patients

[PN: ACROSS, RESPONSE OPTIONS]

1. There is currently no prescription medication for insomnia which is both safe and efficacious for these patients
2. There are significant unmet needs in the current prescription medication options for these types of insomnia patients
3. I am very satisfied with the existing treatment options for these insomnia patients

[PN: SINGLE CODE PER STATEMENT]

E12. Please rank your top 5 **unmet** needs in insomnia treatment.

[PN: RANKING DRAG AND DROP, USE QUESTION LIBRARY FI030]

1. Ability to improve speed of sleep onset
2. Ability to improve sleep maintenance
3. Ability to improve total sleep time
4. Ability to improve sleep **over time**
5. Ability to improve daytime functioning

6. Does not cause morning grogginess
7. Good tolerability
8. Safe for long term use
9. Does not cause dependency
10. Innovative mechanism of action
11. Lower patient out of pocket costs
12. Low risk of drug-drug interactions with commonly taken medication
13. Easier access to CBT-I/ behavioural therapy
14. More objective ways of evaluating treatment efficacy
15. Other (please specify) **[PN: ANCHOR]**
16. I do not have any unmet needs for insomnia treatment **[EXCLUSIVE – ANCHOR]**

[PN: DRAG AND DROP 5 STATEMENTS – SHOW RANKING # IN DROP BOX – ALLOW RESPONDENTS TO CHANGE ORDER IN DROP BOX]
